# Correction: Re-visiting the trans insertion model for complexin clamping

**DOI:** 10.7554/eLife.31512

**Published:** 2017-09-07

**Authors:** Shyam S Krishnakumar, Feng Li, Jeff Coleman, Curtis M Schauder, Daniel Kümmel, Frédéric Pincet, James E Rothman, Karin M Reinisch

Krishnakumar SS, Li F, Coleman J, Schauder CM, Kümmel D, Pincet F, Rothman JE, Reinisch KM. 2015. Re-visiting the trans insertion model for complexin clamping. *eLife* **4**:e04463. doi: 10.7554/eLife.04463.

Published 2, April 2015

1) In the Material and Methods, we found several errors in the plasmid strains used.

The Syntaxin1A protein used for this study was purified from a plasmid that contained Syntaxin1A residues 188 to 265 on a pET28 vector which included an N-terminal 6xHis tag. The previously reported GST-TEV-syntaxin1A (containing rat Syntaxin 1a residues 191–253) was only used in the crystal structure published in Kümmel et al., 2011. In addition, the two helices of SNAP25 were purified from His-SUMO vectors: 6xHis-SUMO rat SNAP25A-N terminal SNARE motif (residues 7-82; SNAP25N), 6x His-SUMO rat SNAP25A-C terminal SNARE motif (residues 141-203; SNAP25C). CPX48-134 was purified using 6xHis-SUMO human CPX1 (residues 48-134), and full length CPX was purified using 6x His-human CPX1 (residues 1-134) on a pET15 vector that contained a Thrombin cleavage site.

The entire 'Plasmid constructs and protein purification' section should be replaced with the following:

“The constructs used in this study are GST-PreScission-VAMP2Δ60 (human VAMP2 residues 29–60), pET15b-oligohistidine-thrombin-VAMP2 (human VAMP2 residues 29–96), pET28-oligohistidine-synataxin1A (containing rat syntaxin 1a residues 191–265), oligohistidine-SUMO-SNAP25N (containing human SNAP25A residues 7–82), oligohistidine-SUMO-SNAP25C (containing human SNAP25A residues 141-203) and pET15b-oligohistidine-thrombin-CPX (containing human complexin1 residues 1-134); residues 1–134 with flexible GPGP insert between residues 49–50 (CPX–GPGP), residues 26–83 (CPX26–83); residues 26–83 with non-clamping mutations A30E, A31E, L41E, A44E (ncCPX26–83). Truncated CPX constructs, including residues 48-134 (CPX-48), residues 26-48 (CPX26-48) was purified as oligohistidine-SUMO constructs. All constructs were expressed and purified as described previously (Krishnakumar et al., 2011; Kümmel et al., 2011). To ensure high quality, all proteins were purified on a High-load Superdex 75 (16/60, GE Healthcare; Piscataway, NJ) gel filtration column.”

2) To further clarify the protocol for the ITC experiment the following information is to be included in the “ITC Analysis” section following the sentence: *To form the blocked SNAREΔ60 complex, purified SNAREΔ60 complex was mixed with 2.5 molar excess of CPX-48 and incubated overnight at 4°C to ensure complete binding.*

“Then, we titrated CPX-48 back into this initial mixture of SNAREΔ60 and CPX48-134. If there was heat signal, we added more CPX-48 to the mixture. This process was repeated until negligible residual heat signal was observed (control experiment). Then we titrated full length CPX1-134 into the exact same mixture of blocked SNAREΔ60 as used in the control experiment. By comparing the full length CPX titration with the control titration, we determined the binding interaction between CPX N-terminus and C-terminal unzippered t-SNARE.”

The article has been corrected accordingly.

